# Chemical composition of surgical smoke produced during the loop electrosurgical excision procedure when treating cervical intraepithelial neoplasia

**DOI:** 10.1186/s12957-021-02211-8

**Published:** 2021-04-09

**Authors:** Yi Liu, Menghuang Zhao, Yongqiang Shao, Linzhi Yan, Xueqiong Zhu

**Affiliations:** 1 Center for Uterine Cancer Diagnosis & Therapy Research of Zhejiang Province, Department of Obstetrics and Gynecology, The Second Affiliated Hospital of Wenzhou Medical University, Wenzhou, 325027 China; 2 Department of Pathology, Beth Israel Deaconess Medical Center, Harvard Medical School, Boston, MA 02215 USA; 3 Wenzhou Center for Disease Control and Prevention, Wenzhou, China

**Keywords:** Chemicals, LEEP, Surgical smoke, Evacuation devices, Healthcare worker

## Abstract

**Background:**

As LEEP (loop electrosurgical excision procedure) is being increasingly used for the diagnosis and treatment of uterine cervical intraepithelial neoplasia, surgical smoke during LEEP has become an inevitable health issue. Therefore, in this study, exposure to the chemical substances in surgical smoke produced during LEEP was assessed.

**Methods:**

Smoke samples from patients with high-grade cervical intraepithelial neoplasia undergoing LEEP were collected by smoke-absorbing devices situated 1 m away from the operating table and near the nose of the operator during LEEP. Each plume sample was collected after 5 patients underwent LEEP, requiring 5 min for smoke collection for each patient. The chemicals of exposure to surgical smoke were assessed, and the hazard classes of these chemical components were evaluated by the International Agency for Research on Cancer.

**Results:**

Qualitative analysis of the smoke produced during LEEP revealed a variety of potentially toxic chemicals under standard detection, such as benzene, toluene, xylene, ethylbenzene, styrene, butyl acetate, acrylonitrile, 1,2-dichloroethane, phenol, chlorine, cyanide, hydrogen cyanide and carbon monoxide. Additionally, the average concentration of carbon dioxide was 0.098 ± 0.015% during surgery and was higher than that before surgery (0.072 ± 0.007%, *P* < 0.001), and the concentration of formaldehyde was significantly higher during surgery (0.023 ± 0.009 mg/m^3^, *P* < 0.05) than before surgery (0.012 ± 0.001 mg/m^3^, *P* < 0.05).

**Conclusions:**

Most of the detected chemical concentrations in smoke generated during LEEP were below the exposure limits when local exhaust ventilation procedures were efficiently used. However, the concentrations of carbon dioxide and formaldehyde found in smoke were significantly higher after surgery. Wearing a high-filtration mask and using evacuation devices routinely and consistently when performing LEEP are recommended to protect perioperative personnel.

**Supplementary Information:**

The online version contains supplementary material available at 10.1186/s12957-021-02211-8.

## Introduction

Surgical smoke is the gaseous byproduct generated from tissues being dissected, excised and coagulated by heat-generating devices such as lasers and electrocautery [1]. During these procedures, the target cells are heated to the point that they start boiling, the cell membranes are ruptured, and subsequently, ultrafine particles are dispersed into the operating theatre [2]. There are mounting disadvantages of surgical smoke, such as obscuring the operating field, producing an unpleasant odour, releasing hazardous chemicals including mutagens and carcinogens, and harbouring contagious, viable malignant cells and even live bacteria and viruses [3, 4]. Each year in America, a total of approximately 500,000 personnel such as surgeons, nurses, anaesthesiologists and technicians are exposed to surgical smoke in operating rooms [5].

Surgical smoke is composed of 95% water vapour and 5% particulate matter [6]. The latter consists of dead and living cellular material, lung-damaging particulates, blood fragments, bacteria, viruses and toxic chemicals [6]. The chemical constituents and amount of noxious smoke vary widely, depending on the type of surgery, type of energy and length of surgery time [7]. So far, in vitro experiments have identified more than 80 chemical compounds in surgical smoke [8]. Emerging evidence has illustrated that exposure to these chemicals can result in harmful effects to surgeons, such as headaches, watery eyes, coughs, burning throats, nausea, bad odours absorbed in the hair, drowsiness, dizziness, sneezing and rhinitis [9].

Cervical cancer is becoming one of the leading causes of cancer-related deaths in women. In 2018 in the USA, an estimated 13,240 patients were diagnosed with this deadly disease, and approximately 4,170 patients died [10]. Therefore, to obtain better treatment outcomes, the loop electrosurgical excision procedure (LEEP) has been considered an accurate technique in cervical cancer prevention for the excision of high-grade cervical intraepithelial neoplasia (CINII and CINIII) [11–13]. However, LEEP uses a low-voltage, high-frequency alternating current of electricity through a thin wire loop to complete surgery, resulting in the generation of a smoke plume [14]. Unfortunately, the operating room personnel are exposed to toxic chemicals from the beginning to the end of the procedure, which is regarded as an occupational hazard to the staff in the operating theatre. To date, there have been no available reports focusing on the potential dangers associated with exposure to smoke generated during LEEP. Therefore, in this study, smoke samples from patients with high-grade cervical intraepithelial neoplasia undergoing LEEP were collected, and the chemicals of exposure in surgical smoke were detected to explore the chemical components and potential hazards of surgical smoke produced during LEEP.

## Materials and methods

Smoke samples were collected from patients who harboured high-grade cervical intraepithelial neoplasia and underwent LEEP in the outpatient department of the Second Affiliated Hospital of Wenzhou Medical University, China, from May 2017 to December 2018. The operating rooms were equipped with local exhaust ventilation procedures, which were connected to the vaginal speculum through a tube. In addition, a vacuum suction device was used in the operating room. The entire dissection and coagulation during LEEP were performed using monopolar electrocautery (HF-120B, PHILIPS). The energy mode was set at 50 W for cutting and 30 W for coagulation. Ethical approval was obtained from the Research Ethical Committee of the Second Affiliated Hospital of Wenzhou Medical University (approval number: KY2016-01; approval date: Jan 29, 2016; Supplement file). All subjects agreed and provided written informed consent before starting the study.

### Smoke sample collection

Surgical smoke was collected and detected according to the Chinese indoor air quality standard (GB/T 18883-2002) [15] and Chinese national standards (GB/T 160.68-2004, GB/T 160.29-2004, GB/T 18204.2-2014 and GB/T 160.51-2007) [16]. Each plume sample was collected after 5 patients underwent LEEP (the amount of surgical smoke for 5 patients can filled with the collection airbags and can be tested). Nine plume samples were collected to analyse expected volatile organic chemicals such as benzene, toluene, xylene, ethylbenzene, styrene, butyl acetate, hendecane, acetone, acrylonitrile, 1,2-dichloroethane, phenol, chlorine, cyanide, hydrogen, carbon monoxide, carbon dioxide and formaldehyde.

**Fig. 1:**
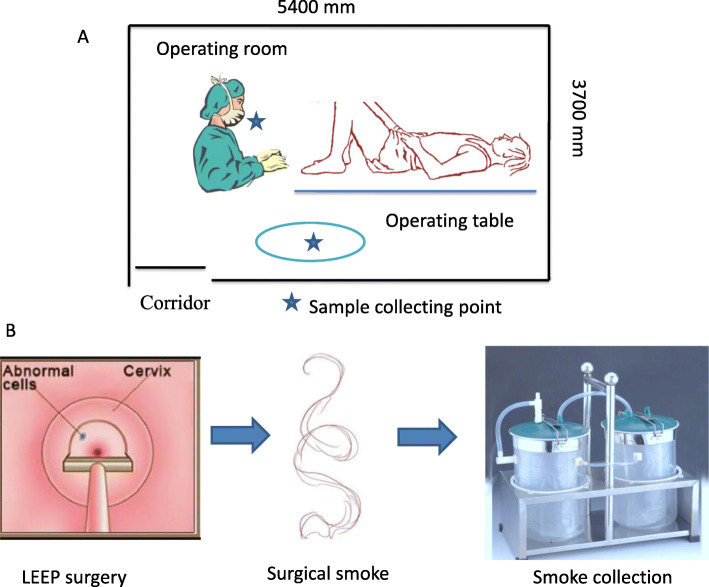
Operating room of LEEP and collection of smoke samples. **a** Operating room for gynaecologist performing LEEP surgery and the collection point of surgical smoke samples. **b** Surgical smoke generated from LEEP surgical was collected by the negative pressure suction device

Smoke samples from before and during LEEP from the same operating room were also collected to minimize background contamination. The collection points were situated 1 metre from the operating table and within the breathing zone of the medical operator (20 cm from the diathermy tip, near the nose of the operator) (Fig. 1a). Smoke samples were collected by activated charcoal tube, absorption liquid, silica gel and microporous filter adsorption and then were collected by a negative pressure suction device (Fig. 1b). Smoke samples were only collected immediately when the electrocautery was activated with a vacuum suction device. The total time for collecting different chemical components was 5 min for every patient. The flow rates used to collect different chemical components varied from 0.1 to 1.0 L/min according to the Chinese national standard, which was set by sampling pumps (GilAir Plus, Sensidyne, USA).

### Analysis of smoke

Analysis was performed at the Wenzhou Disease Control and Prevention Center (Wenzhou, Zhejiang, China). Chlorine, cyanide and hydrogen cyanide in the collected smoke were determined by a barbital sodium-based spectrophotometric method [17]. Chlorine, cyanide and hydrogen cyanide could react with chloramine to form cyanogen chloride in a weakly acidic solution and then react with isonicotinic acid sodium to produce glutaraldehyde acid, which could in turn react with barbituric acid and produce purple compounds. One drop of phenolphthalein solution was added to a standard cyanide solution and neutralized with acetic acid solution. Then, 1.5 ml of buffer and 0.2 ml of chloramine T solution were added for 5 min, followed by 2.5 ml of the coloured solution, and the mixture was placed in a water bath at 25 °C–40 °C for 40 min. Then, the absorbance was determined at 600 nm, reflecting the concentrations of chlorine, cyanide and hydrogen cyanide.

Automated thermal desorption-gas chromatography (Agilent, America) was used to detect benzene, toluene, xylene, ethylbenzene, styrene, butyl acetate, hendecane, acetone, acrylonitrile, 1,2-dichloroethane and phenol. Gas chromatography analysis was performed using an Agilent 789013 gas chromatograph system coupled with a flame ionization detector. An HP-Innowax column (30 m × 0.32 mm × 0.5 μm) was used for chromatographic separations. Thermal desorption was required for the sample to enter the gas chromatography system for the separation step. Thermal desorption of the concentrated compounds was performed at 250 °C for 5 min, during which the eluted volatile organic compounds were transferred to a cold trap maintained at 10 °C. Then, the cold trap was rapidly heated from 10 °C to 250 °C and maintained at this temperature for 3 min. During this time, volatile organic compounds were injected onto the HP-Innowax column. The thermal desorption system transfer line temperature was 150 °C. The gas chromatography conditions were set as follows. The initial temperature was held at 45 °C for 1 min, which was then increased to 85 °C at a rate of 8 °C/min and maintained at 85 °C for 3 min. Finally, the temperature was increased to 200 °C at a rate of 20 °C/min. The flame ionization detector temperature was set at 300 °C. The retention time of the known components in the standard sample was used to identify the compositions of the tested samples. If the retention time of the sample was the same as that of the standard gas under the same gas chromatographic conditions, then the composition in the sample was the same as that in the standard. The peak area was used to quantify the concentration of the compounds in the samples. The ratio of the tested sample peak area to the standard sample peak area was equal to the ratio of the sample concentration to that of the standard gas under the same gas chromatographic analysis conditions.

In addition, carbon monoxide, carbon dioxide and formaldehyde levels were analysed using a portable multi-function tester (Testo 435, Germany) and formaldehyde tester (XP-308B, New-Cosmos, Japan).

### Hazards of smoke

Hazard classes of chemical components were obtained from the International Agency for Research on Cancer (IARC) [18]. Carcinogenic risks to humans have been evaluated by the IARC and classified into 5 groups. Group 1 is carcinogenic to humans, and this category is used in cases of sufficient evidence of carcinogenicity in humans. Group 2A is likely carcinogenic to humans, when there is limited evidence of carcinogenicity in humans and sufficient evidence of carcinogenicity in experimental animals. Group 2B is possibly carcinogenic to humans, and compared to group 2A, group 2B has less than sufficient evidence of carcinogenicity in experimental animals. Group 3 is not classifiable regarding its carcinogenicity to humans, and it is used when the evidence of carcinogenicity is inadequate in humans and inadequate or limited in experimental animals. Group 4 is likely not carcinogenic to humans, lacking evidence of carcinogenicity in humans and in experimental animals.

### Methods of statistical analysis

All of the analyses were performed using SPSS software, version 17.0 (Chicago, IL, USA). Student’s *t* test was performed to compare the differences in volatile chemical compounds before and during the procedures. The level of significance used for the statistical tests was a *P* value < 0.05.

## Results

Among the LEEP smoke samples, the levels of volatile organic chemicals including benzene, toluene, xylene, ethylbenzene, styrene, butyl acetate, hendecane, acetone, acrylonitrile, 1,2-dichloroethane, phenol, chlorine, cyanide, hydrogen cyanide and carbon monoxide were found to be less than the lowest mass of detection (LMD) both in the gas from circumstance and produced during LEEP and vaporization (Table 1). No significant differences were found among the concentrations of these chemicals determined pre- and postoperatively.

**Table 1:** The hazards class and concentration of each common chemical in surgical smoke before LEEP and during LEEP

Chemicals	Hazards class (IARC)	LMD (mg/m^3^)	Air sample before LEEP (mg/m^3^)	Smoke sample during LEEP (mg/m^3^)
Benzene	1	0.01	< 0.01	< 0.01
Toluene	3	0.01	< 0.01	< 0.01
Xylene	3	0.01	< 0.01	< 0.01
Ethylbenzene	2B	0.01	< 0.01	< 0.01
Styrene	2A	0.01	< 0.01	< 0.01
Butyl acetate	3	0.02	< 0.02	< 0.02
Hendecane	NA	0.01	< 0.01	< 0.01
Acetone	NA	0.02	< 0.02	< 0.02
Acrylonitrile	2B	0.02	< 0.02	< 0.02
1,2-dichloroethane	2B	0.02	< 0.02	< 0.02
Phenol	3	0.5	< 0.5	< 0.5
Chlorine	3	0.2	< 0.2	< 0.2
Cyanide	1	0.02	< 0.02	< 0.02
Hydrogen cyanide	1	0.1	< 0.1	< 0.1
Carbon monoxide	NA	0.1	< 0.1	< 0.1

LEEP, loop electrosurgical excisional procedure; LMD, lowest mass of detection; IARC, International Agency for Research on Cancer, 1, carcinogenic to humans; 2A, probably carcinogenic to humans; 2B, possibly carcinogenic to humans, less than sufficient evidence; 3, not classifiable as to its carcinogenicity to humans; NA, not available in IARC

As shown in Table 2, the concentration of carbon dioxide in the surgical smoke during and before LEEP was, on average, 0.098 ± 0.015% and 0.072 ± 0.007%, respectively. The results showed a significantly higher concentration of carbon dioxide during LEEP than before LEEP (*P* < 0.001). In the case of formaldehyde, the average concentration of this compound in the plume during LEEP was also significantly higher than before surgery (0.023 ± 0.009 mg/m^3^ compared to 0.012 ± 0.001 mg/m^3^, respectively *P* < 0.05).

**Table 2:** The hazards class and concentration of carbon dioxide, formaldehyde and carbon monoxide in surgical smoke before LEEP and during LEEP

Chemicals	Hazards class (IARC)	LMD	Before LEEP	During LEEP	*P* value
Carbon dioxide	NA	0.01	0.072 ± 0.007 %	0.098 ± 0.015%	< 0.001
Formaldehyde	1	0.01	0.012 ± 0.001 mg/m^3^	0.023 ± 0.009 mg/m^3^	< 0.05

LEEP, loop electrosurgical excisional procedure; IARC, International Agency for Research on Cancer, 1, carcinogenic to humans; 2A, probably carcinogenic to humans; 2B, possibly carcinogenic to humans, less than sufficient evidence; 3, not classifiable as to its carcinogenicity to humans; NA, not available in IARC; LMD, lowest mass of detection

Chemicals from the smoke samples were further analysed based on the IARC classifications to illustrate their potential hazards. Notably, benzene, cyanide, hydrogen cyanide and formaldehyde were classified as group 1, which is carcinogenic to humans. Styrene was recognized as group 2A, which is likely carcinogenic to humans, and ethylbenzene, acrylonitrile and 1,2-dichloroethane were recognized as group 2B, which is possibly carcinogenic to humans. Furthermore, toluene, xylene, butyl acetate, phenol, and chlorine were recognized as group 3, which is not classifiable for its carcinogenicity to humans. Additionally, hendecane, acetone, carbon monoxide and carbon dioxide were not available in the IARC listings (Tables 1 and 2).

## Discussion

Since electrocautery devices are increasingly used in modern surgery worldwide, surgical smoke has become an inevitable health issue. The potential hazards of surgical smoke have given rise to serious concerns. To date, emerging studies have described the chemical constituents of diathermy plumes produced during various surgeries on live humans. Sagar et al. [19] detected low levels of benzene, styrene, ethyl benzene, carbon disulphide and methyl benzene in the plume generated during colorectal surgery. Hollmann and colleagues identified 11 different gases in surgical smoke collected 2 cm from the tip of the unipolar electrocautery device during reduction mammoplasty [20]. Among these chemical components, 2-furancarboxaldehyde presented a concentration 12 times higher than the occupational exposure limit. Another group collected plumes as close as possible (< 2 cm) to the point of an electrocautery pencil during laparotomy for abdominal surgery and demonstrated that hydrogen cyanide (3–51 parts per million (ppm)), acetylene (2–8 ppm) and 1,3-butadiene (0.15–0.69 ppm) existed in the plume [21]. In addition, emerging evidence has identified the chemical components during transurethral resection of prostates (TURP). One group detected 16 chemical constituents in the gaseous plume collected 15 cm above the end of the resectoscope, in which the concentration of carbon monoxide was found to be significantly high, causing medical operators to experience detrimental side effects such as headache, fatigue and nausea [22]. In line with a previous study, another group also collected 12 smoke samples from TURP and vaporization and identified 16 main chemical constituents in surgical smoke [4]. Later, Lin et al. [23] quantified five volatile organic compounds (toluene, styrene, xylene, phenol and furfural) in smoke collected from the tip of a monopolar electrocauterizer used in mammoplasty. Remarkably, a recent study collected 36 surgical smoke samples using an electrocautery surgical device used in human breast reduction surgeries. They detected 17 different volatile organic compounds, among which acetaldehyde, ethanol and isopropyl alcohol were detected to be highly concentrated in nearly every sample [24]. Similarly, Sigrist et al. [25] identified carbon monoxide, hydrogen fluoride, sevoflurane, methane, ethane, and ethylene during minimally invasive surgery. All these experiments revealed the abundant chemicals in electrocautery smoke. However, most of these volatile organic compounds such as acrolein, acetaldehyde, acrylonitrile, benzene, cyclohexanone, furfural, formaldehyde, polyaromatic hydrocarbons, styrene, toluene and xylene have been classified as carcinogens [26].

Interestingly, harmless concentrations of chemical components have also been detected in some reports. Gianella and colleagues [27] quantitatively evaluated the levels of chemicals in plumes from a vessel-sealing device during laparoscopic surgery and indicated that the concentrations of methane, ethane and ethylene in smoke were lower than the recommended exposure limits. In another study, sample collection during laparoscopic cholecystectomy was performed within the breathing zone of medical staff located near the operating table. Aldehydes, benzene, toluene, ethylbenzene, xylene, ozone, dioxins and furans were identified. However, the levels of all of those compounds were less than the hygienic standards allowed by the European Union Maximum Acceptable Concentration [28].

This study is the first to evaluate the chemical composition of smoke produced during LEEP. Higher concentrations of formaldehyde and carbon dioxide were identified during LEEP than before LEEP in this study. Other toxic compounds such as benzene, toluene, xylene, ethylbenzene, styrene, butyl acetate, hendecane, acetone, acrylonitrile, 1,2-dichloroethane, phenol, chlorine, cyanide, hydrogen cyanide and carbon monoxide detected in the present study were observed at levels less than the LMD.

High concentrations of carbon dioxide have been well documented to have direct health effects on humans [29]. The maximum acceptable indoor carbon dioxide concentration is 800 ppm (a 0.08% concentration) [30]. When the concentration is higher than 20,000 ppm (a 2% concentration), carbon dioxide can cause deepened breathing. When higher than 40,000 ppm (a 4% concentration), carbon dioxide can markedly increase respiration. When higher than 100,000 ppm (a 10% concentration), it can cause visual disturbances and tremors and loss of consciousness. In addition, 250,000 ppm (a 25% concentration) of carbon dioxide can cause death [29]. However, the concentration of carbon dioxide in the surgical smoke during LEEP was detected to be on average 0.098 ± 0.015%, beyond the maximum acceptable indoor carbon dioxide level (0.08%), which was significantly higher than that before LEEP. This result may be attributed to the close quarters in the operating room and leakage from loose masks, suggesting that surgeons in these circumstances should adopt useful methods to minimize carbon dioxide exposure, such as increasing the air cleaning capacity of the operating room, reducing unnecessary surgical staff and wearing tight-fitting masks.

Formaldehyde is classified as a known human carcinogen. The threshold value for indoor formaldehyde concentrations is 0.1 mg/m^3^ [31]. Short-term exposure can result in eye irritation, nausea, vomiting, headache, weakness, oedema, dizziness, fatigue and chest tightness [32]. Long-term exposure in humans may be linked to higher incidences of cancer such as leukaemia [33] and can even result in foetal malformations in pregnant women [32]. Our results showed that the average concentrations of formaldehyde in the surgical plume during LEEP were significantly higher than those before surgery. Although the concentration of formaldehyde was less than the exposure limit (0.1 mg/m^3^), attention should be paid and preventive measures should be undertaken for gynaecologists and other operating room staff members to minimize contact with formaldehyde and to prevent excessive exposure. Moreover, the majority of the previous studies concerning the composition of surgical smoke collected smoke samples near the tip of the diathermy pencil [20, 23]. In our study, surgical smoke was collected from the same gynaecologist who has more than 10 years of experience in LEEP surgery. The gas concentration was measured directly at the height of the noses of gynaecologists, and it was diluted by air. Additionally, the elevated concentration of formaldehyde might have been due to the carbonization of cells, proteins and other organic matter produced by the high temperature from LEEP knife burning. However, the gas concentrations measured within the breathing zone exactly reflected the degree of chemical exposure to operators in the operating room. Therefore, it is necessary for operating room staff to wear high-filtration masks and to have an equipped high-efficiency filtering system in the operating room. Additionally, reducing unnecessary cell burning and performing surgery with the most suitable power are excellent methods to protect personnel from the hazards of formaldehyde.

In addition, emerging evidence has shown that suction devices are often ignored by operators. For example, a web-based survey examined current surgical smoke practices of local exhaust ventilators. The researchers found that only 14% of 4533 respondents reported that local exhaust ventilators were always used during electrosurgery procedures [34]. It is indispensable that suction devices be used in the hospital and execute their protective functions. Suction clearance of the diathermy plume with smoke extraction systems was reported to result in a significant reduction in the amount of smoke in thyroid surgery [35]. With the use of wall suction, Wang et al. [36] found that fine particle inhalation significantly decreased by 48% in superficial surgeries, 52% in abdominal surgeries and 65% in pelvic surgeries. In our experiment, suction devices were effectively used in the operating room, resulting in negative consequences. Therefore, we recommended that practitioners use suction systems routinely and consistently when performing LEEP.

In addition, Zhao et al. [37] identified 39 types of gases generated during transurethral resection of malignant bladder tumour tissues and only 16 types of gases generated during transurethral resection of benign hypertrophic prostate. The differences in the types of gases between benign hypertrophic prostate and malignant bladder tumour tissues indicated that electrosurgery of malignant tissue was likely more hazardous. LEEP is usually used to treat cervical high-grade CIN, which is potentially premalignant [38]. Thus, we suspected that the concentrations of chemical components in smoke produced during LEEP might be less than the levels produced during cervical cancer surgery. The potential hazard of gases during cervical cancer surgery must be further investigated.

Finally, the most important thing is to ensure the health and safety of operating room staff and patients. It is necessary for medical staff exposed to surgical smoke to understand the relevant knowledge and raise awareness of prevention. At the same time, the introduction, usage and management of various protective equipment should be strengthened to reduce the harm of surgical smoke, provide a healthy environment for healthcare workers in the operating room and improve the physical and mental health of the operating room staff. There are some recommendations. First, a high-filtration mask should be used. Usual surgical masks cannot effectively filter out surgical smoke or cannot even be closed to the face in actual use. Therefore, surgical masks that are highly protective against surgical smoke, such as high-filtration masks and N95 respirators, should be worn, which should conform to the shape of the face when worn, fasten to the face and have no gaps around the nose and mouth. In addition, goggles are necessary to protect the eyes. Second, reasonable smoke extraction should be established. During surgical operations, surgical smoke extraction equipment with a high-efficiency filtering system must be operated. When surgical smoke is generated, smoke should be extracted as far away as possible from the surgical area, and this process should be continuous. Third, we must reduce the production of surgical smoke. During operations, electrosurgical knives that produce less burnt smoke and exhibit high performance should be used as much as possible, the modes required for various operations should be adjusted correctly and the knives should be cleaned in time to reduce the generation of surgical smoke. In addition, minimizing unnecessary tissue burning during surgery could decrease the risk of tissue necrosis and infection. Finally, we must improve protection knowledge. Medical staff working in the operating room, including nurses, surgeons and anaesthesiologists, all must be re-educated in knowledge and information about smoke prevention in the operating room. It should be ensured that operating room staff has the ability to use smoke exhaust equipment correctly and to comply with relevant rules and regulations.

## Conclusion

In summary, the present investigation showed relatively low levels of the most common chemical compounds in the smoke from LEEP. In contrast to the levels of these determined compounds, the average concentrations of carbon dioxide and formaldehyde in the plume were significantly higher during LEEP than before it. Although exposure of the patient in the operating room to emerging chemical compounds is usually a one-time and short-term incident, awareness should be strengthened and preventive measures should be implemented for surgeons who frequently perform operations in the operating room in order to protect themselves from long-term exposure. We recommend that practitioners wear high-filtration masks and use evacuation devices routinely and consistently when performing LEEP.

## Supplementary Information

**Additional file 1.**

## Data Availability

All data generated or analysed during this study are included in this published article.
