# Supplementary figures and images for: Chemical composition of surgical smoke produced during the loop electrosurgical excision procedure when treating cervical intraepithelial neoplasia
Source: World J Surg Oncol. 2021 Apr 9;19:103. doi: 10.1186/s12957-021-02211-8 (PMC8034107; doi:10.1186/s12957-021-02211-8)

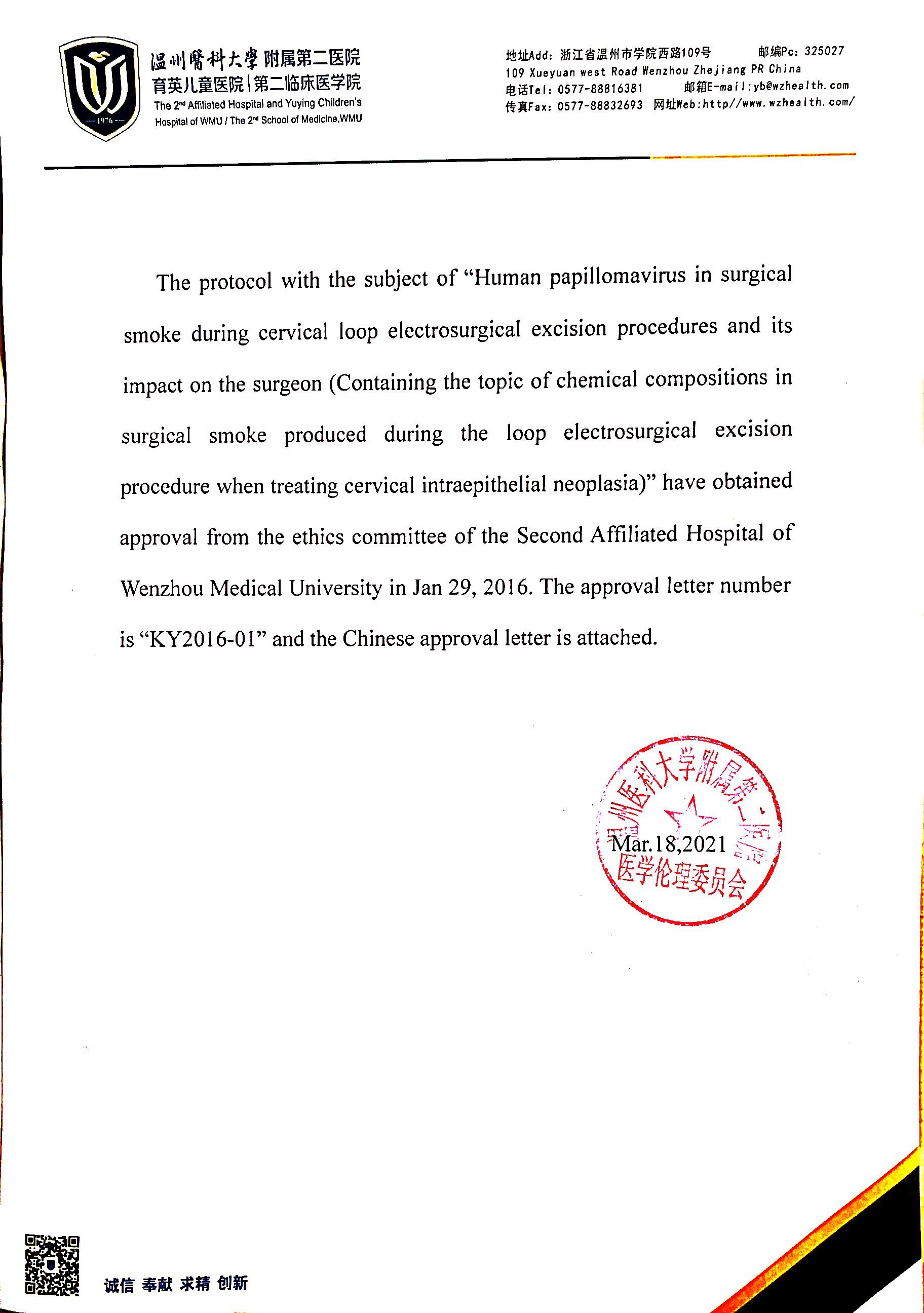

Supplement: Supplementary file 1 — Additional file 1. [file 12957_2021_2211_MOESM1_ESM.jpg]
